# Relationship between fruit and vegetable consumption and internet addiction with insomnia and depression as multiple mediators during the COVID-19 pandemic: a three-wave longitudinal study in Chinese college students

**DOI:** 10.1186/s12888-023-05415-2

**Published:** 2023-12-13

**Authors:** Liqing Yao, Kaixin Liang, Liuyue Huang, Xinli Chi

**Affiliations:** 1 School of Pharmacy, Guangdong Pharmaceutical University, Guangzhou, China; 2 Faculty of Medicine, Medical Sciences Division, Macau University of Science and Technology, Macau, China; 3 School of Psychology, Shenzhen University, Shenzhen, China; 4 The Shenzhen Humanities & Social Sciences Key Research Bases of the Center for Mental Health, Shenzhen University, Shenzhen, China; 5 Department of Psychology, Faculty of Social Sciences, University of Macau, Macau, China

**Keywords:** Fruit and vegetable consumption, Internet addiction, Insomnia, Depression, Longitudinal study, Multiple mediation model

## Abstract

**Background:**

The relationships between fruit and vegetable consumption (FV) and Internet addiction (IA) in college students still remained unknown together with the internal mechanisms. Given the limitations of previous cross-sectional design, longitudinal research was necessary to be conducted to explore more precise correlations. Using the three-wave data in a longitudinal design, this study aimed to explore the association between FV and IA among Chinese college students and potential multiple mediators of insomnia and depression during the COVID-19 pandemic.

**Methods:**

A total of 579 college students were recruited during three waves (T1: August 2020; T2: November 2020; T3: February 2021). FV (T1), insomnia (T2), depression (T2) and IA (T3) symptoms were reported. The descriptive statistics of the sociodemographic characteristics and correlation analyses of the study variables were calculated. The significance of the mediation effects was measured conducting a bootstrap method with SPSS PROCESS macro.

**Results:**

FV was negatively correlated with IA, and lower FV predicted higher risk of IA. Depression mediated the association between FV and subsequent IA. Insomnia and depression were multiple mediators, which in turn mediated the links between FV and subsequent IA.

**Conclusions:**

The three-wave longitudinal study has revealed that FV had indirect effects on IA through individual mediating factor of depression and multiple mediating roles of insomnia and depression sequentially. The policy makers, educators and researchers should pay attention to the impact of the interventions from healthy diet, in order to optimize the coping strategies for preventing college students from IA.

## Background

The COVID-19 pandemic has pervasive influence on addictive behaviors among college students, especially increases the risk of Internet addiction (IA) [1, 2]. During the COVID-19 pandemic, students have to study online at home due to preventative measures (e.g., quarantine, lockdown and social distancing), which may increase the incidence and prevalence of IA [2, 3]. Growing evidence presents that IA can have profound effects on youth development, such as declined physical health, interpersonal difficulties, and poor academic achievement [4, 5]. Chinese college students tend to have fewer compulsory academic tasks and less academic pressure than younger students, which renders them have more free time and more likely to spend too much time accessing the Internet [6]. Additionally, college students have greater developmental and psychological needs than junior and senior high school students, such as the need to set up a sense of identity and develop meaningful intimate relationships, also causing college students to become a particularly vulnerable youth group for IA [6, 7]. Previous meta-analyses demonstrated that the prevalence of IA among Chinese college students was approximately 11.0% [8], which was about 1.57 times higher than that of the general population group (7.02%) [9]. The prevalence of IA among Chinese college students might even persistently increase in the future due to the development of informatization and Internet popularization in daily life. Since IA is a serious problem among college students, it is necessary to explore the predictors of IA in this age group, especially during the COVID-19 pandemic.

A healthy lifestyle may be a key determinant in preventing Internet addiction (IA) [10–12]. Research indicates that healthy lifestyle behaviors, including nutritious eating, contribute significantly to mental health. Not only do they help in reducing emotional problems, but they are also associated with preventing addictive disorders, including IA. This connection underscores the importance of integrating high nutritional quality and healthy eating behaviors into daily life as a strategy for IA prevention [13, 14]. It is known that fruit and vegetable consumption (FV) should be regarded as important components of healthy eating habits [15]. Low FV is a recognized modifiable risk factor contributing to the rising burden of physical, psychological, and behavioral health problems [16, 17]. Studies have shown that increasing FV consumption can be an effective prevention and treatment strategy for Internet addiction (IA) and other psychological issues, such as depression and anxiety [18, 19]. Fruits and vegetables, rich in vitamin C, B vitamins, and complex carbohydrates, promote the synthesis of mood-related neurotransmitters like dopamine [20], which is linked to higher levels of well-being [20] and can prevent problematic behaviors, including IA. Therefore, FV intake could be a key predictor of IA among Chinese college students. However, related studies that the association between FV and IA still remain scanty in this population group. Besides, empirical validation combined with the background of COVID-19 pandemic is warranted. During the COVID-19 pandemic, it was reported that individuals decreased their consumption of any type of fruits and vegetables because of poor quality and availability, high price, reduced store trips, and the concerns of contamination [21], and the pandemic-related stress has already impacted nutrition behaviors among the college students at the risk of poor FV and even food insecurity [22]. More importantly, despite that previous findings indicate that low consumption of fruits and vegetables may be significantly related to high risk of IA, the underlying mechanisms (i.e., how FV influences IA) remain unclear, especially in Chinese college students. FV is proved to affect mental health [17], and mental health problems are also found that can influence IA [23, 24]. Hence, mental health problems, such as insomnia and depression, may shed insights to our understanding of the mechanisms underlying the relationship between FV and IA.

Insomnia can be a possible mediator in the relationship between FV and IA. Recently, research evidence has revealed high prevalence of insomnia in varying degrees among Chinese college students (16.9%) during the COVID-19 pandemic [25]. Regarding FV and insomnia, a prospective cohort study and another study with a large sample size of university students from 28 countries demonstrated that high FV could relieve insomnia symptoms and promote sleep quality [26, 27]. Additionally, a systematic review including 29 studies also confirmed that the nutritive value of fruits and vegetables might beneficially affect the circadian rhythm leading to positive modification in sleep [28]. On the other hand, regarding insomnia and IA, researchers have stressed that increased levels of insomnia symptoms were associated with an increased risk of IA. A previous study demonstrated that insomnia was very common among Chinese college students with IA [29]. Additionally, evidence from a systematic review reported that participants who had experienced insomnia were about 1.5 times as likely to be a problematic Internet user in comparison to those without insomnia symptoms [30]. Thus, drawing from the aforementioned research, FV might be related to IA through insomnia.

Depression, as a common mental health problem characterized by negative affect, may serve as an additional underlying mediator which is also important. Recently the overall prevalence of depression ranged from 12.2 to 29.7% in Chinese college students during the COVID-19 pandemic [31, 32]. Numerous studies have shown that there is an inverse association between FV and depression [33, 34]. For instance, a meta-analysis with the data from 25 low- and middle-income countries reported that increased FV was related to a lower risk of depression in young people [35]. Other studies also presented that the presence of high levels of antioxidants in fruits and vegetables could dampen some of the detrimental effects of oxidative stress to protect against depression [36, 37]. Additionally, depression is a key predictor of IA. According to the secondary psychiatric disorder hypothesis, psychiatric disorders contributed to the development of addictive behaviors [38]. From the perspective of IA, a longitudinal study suggested that anhedonia (i.e., difficulty experiencing pleasure, a key facet of depression) might contribute to the development of IA in the emerging adult population [39]. Individuals with psychological problems and poor interpersonal relationships preferred online interaction and used the internet to cope with loneliness, and this proclivity could increase the risk of developing IA [40, 41]. Given the observed associations between specific eating patterns, depression and IA, it is necessary to investigate the potential effects of FV on IA through depression. Hence, simultaneously, it was expected that FV might be related to IA through depression.

As a kind of sleep disturbance, insomnia has been revealed that often precedes the onset of an episode of depression in clinical and epidemiological research [42–44]. Evidence from a meta-analysis demonstrated that individual with insomnia may increase the risk of depression [45], and insomnia can also influence the course of depression treatment [46]. It was indicated that disturbances in circadian rhythms caused by insomnia may affect emotional regulation function, causing mood disturbances and even depression [47]. Additionally, according to the neuroplasticity hypothesis, it was found that chronic sleep disruptions in the form of insomnia may increase vulnerability for a dysfunction of neuroplasticity [45, 48]. That is, insomnia may disrupt synaptic plasticity and neural network function then can lead to depression [47]. Thus, insomnia can affect depression symptoms. Taken together, combining the associations among insomnia, depression and IA, it could be expected that insomnia might influence IA by increasing depression symptoms. According to biopsychosocial model, high FV might prevent comorbid psychological disorders including insomnia and depression through physiological mechanisms (e.g., FV-rich diets are conducive to increasing levels of brain-derived neurotrophic factor and contribute towards good mental health), then the low symptoms of comorbid affective disorders can be beneficial to the low levels of social behavioral problems including IA [49–51]. Based on the theoretical exposition and literature review above, insomnia and depression might sequentially participate in influencing mechanism pathways of FV on IA.

**Fig. 1:**
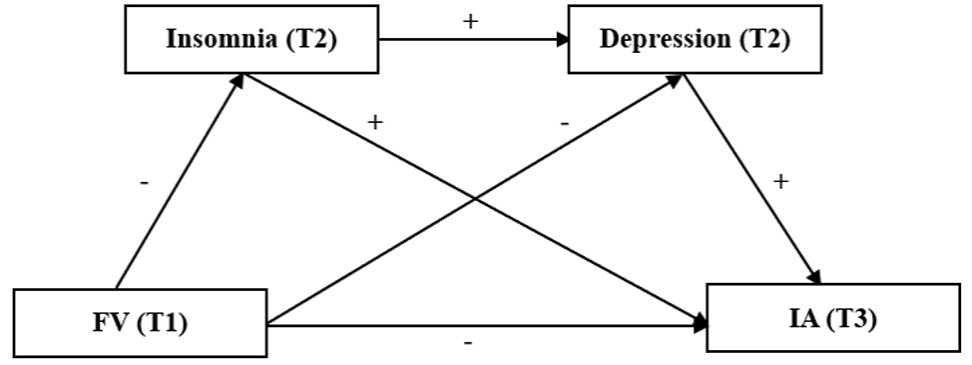
The hypothesized model Note: FV = fruit and vegetable consumption, IA = Internet addiction, T1 = time 1, T2 = time 2, T3 = time 3

Collectively, there is little research focusing on multiple mediating mechanisms of insomnia and depression in the relationship between FV and IA among Chinese college students. Firstly, evidence concerning about sedentary behaviors and dietary intakes has indicated that longer total screen and sitting times are associated with higher prevalence of low FV [52]. Secondly, due to the impact of the COVID-19 pandemic, the eating habits and behaviors have significant changes [53, 54], and the incidence and prevalence of IA, insomnia and depression symptoms have increased among the college students [55, 56]. However, whether and how these variables are related has not been explored, which will help to provide empirical evidence and suggestions for the development of targeted intervention plans in the future. Therefore, it is imperative to investigate the potential pathways and mechanisms underlying these relationships in the context of the pandemic. Besides, most of the previous studies in related fields are cross-sectional design [19, 57], and longitudinal studies with multiple-time measurements and stronger causal explanations are still lacking. Accordingly, based on the theories and empirical studies above, we performed a longitudinal study, analyzed three waves of data, and conducted a multiple mediation model to test the mediating roles of insomnia and depression in the relationship between FV and IA among Chinese college students during the COVID-19 pandemic. The hypotheses addressed were: (1) Low frequency of FV would be associated with high severity of IA; (2) insomnia would mediate the relationship between FV and IA; (3) depression would mediate the relationship between FV and IA; and (4) insomnia and depression might sequentially mediate the relationship between FV and IA (Fig. 1). The findings of the multiple mediation model in the longitudinal study would help to understand how FV predicted IA in college students, so as to provide implications for effective prevention and intervention strategies against IA and other mental health problems among this population group in public emergencies such as the COVID-19 pandemic.

## Methods

### Participants and procedure

The longitudinal study was performed from August 2020 to February 2021 during the COVID-19 pandemic in China. A snowballing sampling method was used to recruit college students in China. The participants were recruited from Chinese social platforms (e.g., WeChat and Tencent QQ), and were invited to complete a survey via a Chinese online survey platform (https://www.wjx.cn). All the participants signed the informed consent before completing the questionnaires.

Data were collected in three waves with three-month intervals: August 2020 (T1), November 2020 (T2), February 2021 (T3). Specifically, FV was measured at T1, insomnia and depression were measured at T2, and IA was measured at T1 and T3. The research also collected demographic information including age, gender, family structure, subjective socioeconomic status (SES) and body mass index (BMI) at baseline. 1162 college students provided valid information in the baseline survey at T1 and 852 (73.3%) participants remained at T2. At T3, a total of 579 (49.8%) participants, whose phone numbers were matched with those in both previous waves, were included in the final sample and analysis. The project was approved by the Human Research Ethics Committee of the Shenzhen University, and all methods were carried out in accordance with relevant guidelines and regulations.

### Measures

#### Sociodemographic information

Participants reported their sociodemographic characteristics including age, gender, family structure, SES and BMI at baseline.

#### IA

The Chinese version of Young’s 10-item Internet Addiction Test (IAT) was used to assess the severity of IA [58]. It contained 10 items such as, “Do you need to spend increasing time on the Internet to get satisfaction?” Participants selected the options of “0 = No” or “1 = Yes” in this 2-point Likert-type scale according to their reality. Total scores ranged from 0 to 10 in this study, and higher total scores indicated a greater severity of IA symptoms. The Internet Addiction Test (IAT) was selected for several reasons: (1) It’s a well-established tool and among the most widely utilized scales for assessing IA issues [5, 59–61]. (2) The IAT measures generalized IA, rather than specific forms like Internet gaming addiction, enabling a more comprehensive assessment of IA [5, 9]. (3) It has demonstrated strong reliability and validity in studies involving Chinese youth [58, 62]. The Cronbach’s α values were 0.823 at T1 and 0.827 at T3 in this study.

#### FV

Two items from the nutrient sub-scale of the Chinese version of the Health Promoting Lifestyle Profile-II (HPLP-II) was used to measure FV (i.e., “Daily fruit consumption” and “Daily vegetable consumption”) [63]. Participants reported their frequency of intaking fruits and vegetables according to a 4-point Likert-type scale (1 = Never to 4 = Routinely). Total scores ranged from 2 to 8, and higher total scores indicated higher level of FV. The HPLP-II has presented good reliability and validity in Chinese young people [64]. The Cronbach’s α coefficients of the HPLP-II in this study were 0.752 at T1.

#### Insomnia

The Chinese version of the Youth Self-Rating Insomnia Scale (YSIS) was utilized to evaluate participants’ insomnia symptoms [65] and the scale has been proven to have good validity and reliability. There were 8 items on a 5-point Likert-type scale, participants were required to report their feelings over the past one month (e.g., “Do you have a sleep problem with sleep insufficiency?”). Total scores that ranged from 8 to 40 can be calculated, and higher total scores indicated a greater severity of insomnia symptoms. In this study, the Cronbach’s α coefficient of the YSIS at T2 was 0.868.

#### Depression

The Patient Health Questionnaire-9 (PHQ-9) was gotten to measure the severity of depression symptoms [66, 67]. It contained 9 items such as “Feeling down, depressed, or hopeless over the past two weeks.” All items were scored according to a 4-point Likert-type scale with the frequencies of the feelings from the participants (0 = Not at all to 3 = Nearly every day). All items were summed up and total scores ranged from 0 to 27, with higher total scores indicating a greater severity of depression symptoms. The Chinese version of the PHQ-9 has also been proven to have good validity and reliability [68]. The Cronbach’s α coefficient of the PHQ-9 at T2 was 0.883 in this study.

### Statistical analysis

IBM SPSS 26.0 was used for conducting descriptive statistics of the sociodemographic characteristics and correlation analyses of the study variables. The multiple mediation model was then tested using the PROCESS macro in SPSS (Model 6) developed by Hayes [69]. The mediation analyses were performed using one independent variable (FV at T1), one dependent variable (IA at T3), two mediators (insomnia and depression at T2). Age, gender, family structure, SES, BMI and IA symptoms at baseline (T1) were included in the model as covariates. The bootstrap method with 5,000 resamples was carried out to measure the indirect effect and to obtain bootstrap confidence intervals of parameter estimation. The bootstrap method has been found to have high power when maintaining reasonable control over the Type I error rate [70], and has been widely known and implemented [70–72]. The regression coefficients were mainly standardized in the results. 95% confidence intervals were estimated in the analyses. The effect was considered significant if a confidence interval did not include zero [73].

## Results

### Sociodemographic characteristics of participants

A total of 579 college students were included in the data analyses in this longitudinal study. The average age was 20.76 years (SD = 1.71) at baseline and the percentage of female was 66.3%. As for the family structure, 89.8% of the participants reported intactness. The mean value of SES was 4.78 (SD = 1.51). The BMI at baseline was 20.14 kg/m^2^ (SD = 2.65) in average. More details were presented in Table 1.

**Table 1:** Sociodemographic characteristics of participants at baseline (N = 579)

Characteristics	n/M	%/SD
**Age (years)**	20.76	1.71
**Gender**		
Male	195	33.7%
Female	384	66.3%
**Family structure**		
Intactness	520	89.8%
Non-intactness	40	6.9%
Other	19	3.3%
**SES (points)**	4.78	1.51
**BMI (kg/m** ^**2**^ **)**	20.14	2.65

Note: M = Mean, SD = standard deviations, SES = subjective socioeconomic status, BMI = body mass index

### Preliminary correlation analyses

Spearman correlation analyses were performed since some of the data were not normally distributed. The results indicated that FV (T1) was negatively correlated with IA (T3), insomnia (T2), depression (T2), and IA (T1); IA (T3), insomnia (T2), depression (T2), and IA (T1) were positively correlated with each other. The means, standard deviations and the results of correlation analyses in detail were all shown in Table 2.

**Table 2:** Spearman correlation analysis of variables

Variables	M	SD	1	2	3	4	5
1. FV (T1)	6.19	1.35	1				
2. IA (T1)	4.16	2.88	-0.181***	1			
3. Insomnia (T2)	18.69	6.45	-0.185***	0.325***	1		
4. Depression (T2)	6.26	4.47	-0.216***	0.427***	0.604***	1	
5. IA (T3)	3.90	2.87	-0.180***	0.615***	0.307***	0.436***	1

Note: M = Means, SD = standard deviations, FV = fruit and vegetable consumption, IA = Internet addiction, T1 = time 1, T2 = time 2, T3 = time 3. *p < 0.05, **p < 0.01, ***p < 0.001

### Analyses of the multiple mediation model

Following the results of correlation analyses, multiple mediation analyses were performed to further examine the associations among FV (T1), insomnia (T2), depression (T2), and IA (T3) in college students. After controlling for age, gender, family structure, SES, BMI, and IA (T1), FV (T1) negatively predicted insomnia (T2) (β = -0.134, *p* < 0.01, 95% CI = -1.017 to -0.261) and depression (T2) (β = -0.078, *p* < 0.05, 95% CI = -0.471 to -0.042) respectively. Moreover, depression (T2) positively predicted IA (T3) (β = 0.230, *p* < 0.001, 95% CI = 0.095 to 0.200). Insomnia (T2) also had a significant relationship with depression (T2) (β = 0.500, *p* < 0.001, 95% CI = 0.300 to 0.392) (Table 3).

**Table 3:** Mediating analysis of FV (T1) and IA (T3)

Outcome variable	Predictive variable	B	SE	β	t	LLCI	ULCI	R^2^
*Direct effect*								
Insomnia (T2)	FV (T1)	-0.639	0.192	-0.134	-3.319**	-1.017	-0.261	0.132***
	Age	0.049	0.149	0.013	0.330	-0.244	0.343	
	Gender	0.658	0.561	0.048	1.174	-0.443	1.760	
	Family structure	0.347	0.592	0.023	0.586	-0.815	1.509	
	SES	-0.227	0.173	-0.053	-1.313	-0.566	0.112	
	BMI	-0.157	0.100	-0.065	-1.577	-0.353	0.039	
	IA (T1)	0.639	0.090	0.285	7.085***	0.462	0.816	
Depression (T2)	FV (T1)	-0.257	0.109	-0.078	-2.352*	-0.471	-0.042	0.430***
	Insomnia (T2)	0.346	0.024	0.500	14.732***	0.300	0.392	
	Age	0.156	0.084	0.060	1.860	-0.009	0.321	
	Gender	0.485	0.315	0.051	1.538	-0.135	1.104	
	Family structure	-0.006	0.332	-0.001	-0.017	-0.659	0.647	
	SES	-0.255	0.097	-0.086	-2.626**	-0.446	-0.064	
	BMI	-0.007	0.056	-0.004	-0.116	-0.117	0.104	
	IA (T1)	0.343	0.053	0.221	6.497***	0.239	0.447	
IA (T3)	FV (T1)	-0.116	0.070	-0.055	-1.654	-0.254	0.022	0.437***
	Insomnia (T2)	-0.001	0.018	-0.001	-0.028	-0.035	0.034	
	Depression (T2)	0.147	0.027	0.230	5.508***	0.095	0.200	
	Age	-0.030	0.054	-0.018	-0.564	-0.136	0.075	
	Gender	0.558	0.202	0.092	2.763**	0.161	0.955	
	Family structure	-0.027	0.212	-0.004	-0.125	-0.444	0.391	
	SES	0.055	0.062	0.029	0.876	-0.068	0.177	
	BMI	0.093	0.036	0.085	2.581*	0.022	0.163	
	IA (T1)	0.508	0.035	0.510	14.526***	0.440	0.577	
*Total effect*								
IA (T3)	FV (T1)	-0.186	0.071	-0.088	-2.604**	-0.326	-0.046	0.395***
	Age	-0.005	0.055	-0.003	-0.087	-0.114	0.104	
	Gender	0.663	0.208	0.109	3.184**	0.254	1.072	
	Family structure	-0.010	0.220	-0.001	-0.045	-0.441	0.422	
	SES	0.006	0.064	0.003	0.088	-0.120	0.132	
	BMI	0.084	0.037	0.077	2.261*	0.011	0.156	
	IA (T1)	0.591	0.033	0.593	17.660***	0.525	0.657	

Note: SE = standard error, CI = confidence interval, LLCI = low limit confidence interval, ULCI = upper limit confidence interval, FV = fruit and vegetable consumption, IA = Internet addiction, SES = subjective socioeconomic status, BMI = body mass index, T1 = time 1, T2 = time 2, T3 = time 3. *p < 0.05, **p < 0.01, ***p < 0.001

**Fig. 2:**
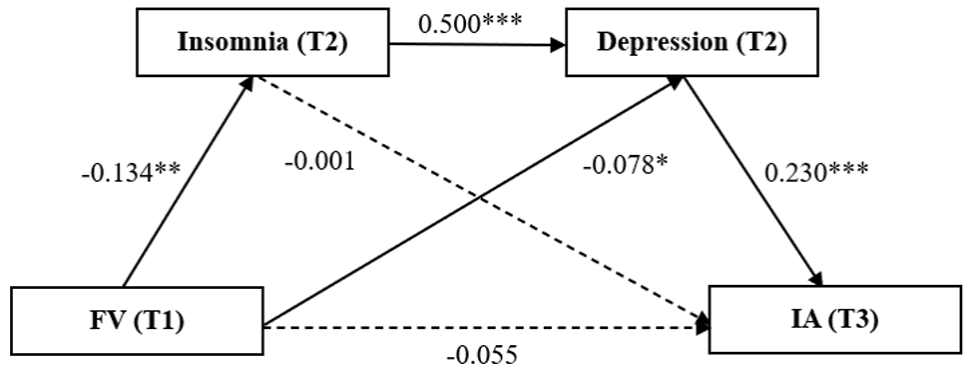
Multiple mediation model about the effect of FV on IA. Note: FV = fruit and vegetable consumption, IA = Internet addiction, T1 = time 1, T2 = time 2, T3 = time 3. Controlling for age, gender, family structure, SES, BMI and IA (T1). *p < 0.05; **p < 0.01; ***p < 0.001

To confirm the significance of the indirect effects of FV through insomnia and depression, bootstrapping method with 5,000 samples was performed at the 95% confidence interval (CI). The results presented that the indirect effect of FV (T1) significantly had an impact of -0.038 that was produced by depression (T2) as a mediator on IA (T3) (95% boot CI = -0.081 to -0.003), with bootstrap 95% confidence interval being not overlapping with zero, and accounting for 20.4% of the total effect. The indirect effect of FV (T1) significantly had an impact of -0.033 that was produced by insomnia (T2) and depression (T2) as mediators on IA (T3) in sequence (95% boot CI = -0.059 to -0.012), accounting for 17.7% of the total effect. The ratio of total indirect effect to total effect was 37.6% (Tables 3 and 4). Standardized path coefficients for the multiple mediation model were presented in Fig. 2.

**Table 4:** Analysis of indirect effects by bootstrap

Path	Effect	Boot SE	Boot LLCI	Boot ULCI
Total indirect effect of FV (T1) on IA (T3)	-0.070	0.023	-0.120	-0.028
FV (T1) → Insomnia (T2) → IA (T3)	0.000	0.012	-0.022	0.025
FV (T1) → Depression (T2) → IA (T3)	-0.038	0.019	-0.081	-0.003
FV (T1) → Insomnia (T2) → Depression (T2) → IA (T3)	-0.033	0.012	-0.059	-0.012

Note: SE = standard error, CI = confidence interval, LLCI = low limit confidence interval, ULCI = upper limit confidence interval, FV = fruit and vegetable consumption, IA = Internet addiction, T1 = time 1, T2 = time 2, T3 = time 3

## Discussion

In this three-wave longitudinal study on Chinese college students during the COVID-19 pandemic, we examined the influence of FV on IA, and further examined the multiple mediating roles of insomnia and depression on these associations. To our knowledge, this is one of the first studies to examine the roles and pathways of FV, insomnia and depression on IA among Chinese college students during the COVID-19 pandemic. The present study has established a significant association between IA and the determinant of FV, mediated by the presence of insomnia and depression. In order to ameliorate IA among college students during the COVID-19, it is imperative to devise and implement interventions that encourage good eating and sleeping habits, and prevent depression symptoms.

In the correlation analyses and total effect model, this study found that lower FV was more likely to increase the severity of IA symptoms of the individuals during the COVID-19 pandemic. These findings were in line with another study about diet and IA focused on inadequate fruit and vegetable intakes as the risk factors of interest [19]. One of the potential mechanisms of the influence of FV on IA can be explained by neurobiological indicators. Higher FV may modulate the sympathetic and parasympathetic nervous activity to reduce their over activity and help prevent individuals’ emotional reactivity from excessive state, which can make the individuals be rational with the brain reward process when using the Internet and relieve the displeasure when not using the Internet, then alleviate the dependence on the Internet [74–76]. Therefore, it is reasonable that increasing the FV may be helpful to prevent college students from IA.

This study found that lower FV predicted higher severity of insomnia symptoms during the COVID-19 pandemic. This result is consistent with previous research that indicated that higher fruit and vegetable intakes are conducive to sleep health (especially the improvements in insomnia-related sleep difficulties) [77]. Low glycemic index could be one of the characteristics of fruits and vegetables with high fiber content that contribute to reducing insomnia symptoms [26]. In addition, as important constituents of an anti-inflammatory diet, eating fruits and vegetables in high intakes may help improve insomnia symptoms and sleep quality by promoting production of melatonin and other neurotransmitters including onset and maintenance of sleep, together with restoring the circadian rhythm of the master biological clock [77–79]. These mechanisms could be responsible for the findings that higher FV may be beneficial to sleep.

Our study demonstrated that FV could influence IA through depression, although the direct effect of FV on IA wasn’t found. Certain vitamins, antioxidants and minerals are widely present in fruits and vegetables were found to exert influence on the mechanisms of depression. Vitamin C, vitamin E and folate, which are contained in fruits and vegetables and play important roles in the endothelial cell signaling cascades, are confirmed that can dampen the detrimental effects of oxidative stress and inflammation on depression [36, 80, 81]. Moreover, dietary polyphenols that are also present in fruits and vegetables may also play important roles in depression. Polyphenols’ antioxidant properties and biomodulating effects on specific cellular signaling pathways related to synaptic plasticity and neuronal stability may be beneficial to the prevention of depression [17, 82]. It is also worth noting that dietary fiber which is abundant in fruits and vegetables, can prevent the perturbations of gut microbiota stability and diversity, then improve the mental health outcomes (e.g., depression), since gut microbiota has been shown to participate in the modulation of psychological health through the microbiome-gut-brain axis [83–86]. Under the background of COVID-19 pandemic with high prevalence of depression among college students, the positive effect of depression on IA in our study supported the previous findings that individuals with depression tended to use the Internet to seek gratification and relieve negative mood through the Internet according to the self-medication and compensatory Internet use theories, which may compel them to use the Internet more frequently and cause IA problems [87–89]. Furthermore, perceived stress responses to situational factors which result from depressive status, may influence personal cognitive processes, for example, by focusing the attention to risky decision-making and short-term rewards [90, 91]. Perceived stress may potentially impact whether or not depressive individuals decide to cope with the related cognitions and affects by using the Internet, which may increase the risk of addictive behaviors of the Internet [91, 92]. In addition, lower FV especially lower fruit consumption, has been linked with lower socioeconomic status which has been shown to associate with higher risk of depression then could predict IA [34].

It was worth demonstrating that FV was available to predict IA through multiple mediating effects of insomnia and depression successively. Individuals with higher levels of insomnia have caused depressive symptoms, which may lead to IA. This result suggests that low FV may have negative impacts on sleep and fit with the previous findings [34]. Students with negative feelings or even depression are prone to use the Internet for emotion regulation, this behavior is linked to high severity of insomnia symptoms, also supporting the evidence from other studies [93, 94]. The mechanisms that insomnia leads to an increase in depressive symptoms can be explained that sleep deprivation increases blood pressure, proinflammatory cytokines, sympathetic tone and evening cortisol levels, which may result in producing significant increases in depression [95]. Therefore, based on the explanation that insomnia affects depression, together with the discussion related to the direct effect of FV on insomnia, and the individual mediator of depression above, the multiple mediation model that insomnia and depression sequentially mediate the relationship between FV and IA are valid. Our findings also supported the cognitive-behavioral model [96], holding that individuals with healthy eating habits might be prone to reduce the use of the Internet for emotion and sleep regulation. Based on their cognitive levels, they have higher health literacy and know the harm of problematic use of the Internet to manage sleep problems and negative emotions [97, 98]. Additionally, in the present study, insomnia individually failed to mediate the pathway from FV to IA significantly, which highlighted the role of depression as a key mediating factor in the whole model. According to our findings, it is possible that depression is the strongest risk factor for IA in the presence of FV and insomnia. As the risk factors identified in this study can be measured to predict IA, it is suggested that the strategies that improve diet quality (e.g., increasing FV), ameliorate insomnia and depression are necessary to be used, this may be helpful and essential to reduce the situations of overusing the Internet and prevent IA in the college students during the COVID-19 pandemic.

### Strengths and implications

This study has several strengths and significant implications. First, we used to a three-wave longitudinal study design to assess Chinese college students’ eating habits, mental health and IA status across half a year in the period of the COVID-19 pandemic. Compared to cross-sectional design in previous studies, our study design can promote causal inference and more comprehensively investigate the direction of the associations between fruit and vegetable intakes and IA. Second, our study revealed the mediation mechanisms with the multiple mediating effects of insomnia and depression, which can contribute to a better understanding of how FV is linked to IA among college students. Third, our study is of great significance for the prevention and intervention of IA in college students, indicating that improving healthy eating habits may be conducive to reducing the prevalence or incidence of IA. Therefore, relevant educators and nutritionists can take some intervention measures to improve students’ healthy eating habits (such as eating fruits and vegetables routinely). Fourth, according to the critical mediating roles of insomnia and depression, our study also reveals that helping college students improve their sleep quality and relieve depressive emotions will have beneficial effects on weakening IA during the COVID-19 pandemic. Students are necessary to pay attention to developing good sleeping habits and improve their sleep quality under the background of COVID-19, which can not only help improve immunity to prevent the disease, but also can help regulate emotions. While students need to learn how to improve their emotional management abilities, mental health teachers in colleges should also actively implement the related work in order to guide students (especially those who have fear of COVID-19) to reasonably express their emotions.

### Limitations and future studies

The present study also has some limitations. First, since measures were self-reported in this study, there might be self-reporting bias and shared method variance, for example the inherent recall bias regarding food consumption. Future studies can consider employing objective measures to make the results more accurate, such as utilizing mobile applications to record Internet usage time and using cluster analysis to identify groups with IA, which may be also beneficial to capture the nuanced differences in IA. Second, the measurement of FV relied on two questions, which might not accurately reflect the actual intake of individuals. Hence, more accurate and comprehensive dietary measurements, such as Food Frequency Questionnaires or 24-hour recall methods, are recommended to obtain more precise information in future studies. Third, the present study was performed using the snowball sampling method, which might induce sample bias. Future studies need to adopt random sampling or stratified sampling techniques in order to enhance the representativeness and generalizability. Fourth, only 49.8% of the participants completed all three waves of the survey and were included in final analyses, which might cause attrition bias and reduce the statistical power. Hence, it is imperative to explore the possibility of sample selection bias due to missing data, and consider utilizing methods such as multiple imputation to handle the missing data in the future studies. Fifth, although we have included age, gender, family structure, SES, BMI and IA (T1) as covariates, other potential confounders (e.g., physical activity, depression and insomnia at T1, and the change of FV from T1 to T3) that might impact mental health and IA status among college students during the COVID-19 pandemic were not considered in this study. Therefore, to ensure the accuracy of the results, future studies can further adjust other potential variables as well. For example, the change of FV might have the largest impact on the findings, it is necessary to keep dynamic monitoring of dietary changes and control them in the future. Moreover, since our study did not analyze each variable at multiple time points, the cross-lagged model could not be performed. The analyses of cross-lagged model are necessary to be explored in future studies because the associations between variables may be bidirectional.

## Conclusions

This longitudinal study has revealed that FV had indirect effects on IA through individual mediating factor of depression and multiple mediating roles of insomnia and depression sequentially in Chinese college students during the COVID-19 pandemic. Specifically, this study suggests that good eating habits (e.g., intaking more fruits and vegetables) may predict lower symptoms of IA disorder, and the effect was passed through insomnia and depression. According to these findings, policy makers, educators and researchers in administrative roles in dietary and mental health should pay attention to the impact of the interventions from healthy diet (e.g., increasing FV through enhancing curricula and experiential learning approaches which are about improving the preference of FV) on insomnia, depression and IA, in order to optimize the coping strategies for preventing college students from IA and other mental health problems based on the perspective of healthy lifestyle.

## Data Availability

The data analyzed in this study are available from corresponding author on reasonable request.

## List of Abbreviations

FV: Fruit and vegetable consumption
IA: Internet addiction
SES: Subjective socioeconomic status
BMI: Body mass index
T1: Time 1
T2: Time 2
T3: Time 3
M: Mean
SD: Standard deviations
SE: Standard error
CI: Confidence interval
LLCI: Low limit confidence interval
ULCI: Upper limit confidence interval
